# Supervisors’ intention to observe clinical task performance: an exploratory study using the theory of planned behaviour during postgraduate medical training

**DOI:** 10.1186/s12909-020-02047-y

**Published:** 2020-04-30

**Authors:** Laury P. J. W. M. de Jonge, Ilse Mesters, Marjan J. B. Govaerts, Angelique A. Timmerman, Jean W. M. Muris, Anneke W. M. Kramer, Cees P. M. van der Vleuten

**Affiliations:** 1 Department of General Practice, Maastricht University, P.O. Box 616, 6200 MD Maastricht, The Netherlands; 2 Department of Epidemiology, Maastricht University, Maastricht, The Netherlands; 3 Department of Educational Research and Development, Maastricht University, Maastricht, The Netherlands; 4 Department of Family Medicine, Leiden University, Leiden, The Netherlands

**Keywords:** Assessor cognition, Attitude of health personnel, Clinical competence, Competency based medical education, Education, medical, graduate, Observations, Theory of planned behaviour, Workplace-based assessment and learning

## Abstract

**Background:**

Direct observation of clinical task performance plays a pivotal role in competency-based medical education. Although formal guidelines require supervisors to engage in direct observations, research demonstrates that trainees are infrequently observed. Supervisors may not only experience practical and socio-cultural barriers to direct observations in healthcare settings, they may also question usefulness or have low perceived self-efficacy in performing direct observations. A better understanding of how these multiple factors interact to influence supervisors’ intention to perform direct observations may help us to more effectively implement the aforementioned guidelines and increase the frequency of direct observations.

**Methods:**

We conducted an exploratory quantitative study, using the Theory of Planned Behaviour (TPB) as our theoretical framework. In applying the TPB, we transfer a psychological theory to medical education to get insight in the influence of cognitive and emotional processes on intentions to use direct observations in workplace based learning and assessment. We developed an instrument to investigate supervisors intention to perform direct observations. The relationships between the TPB measures of our questionnaire were explored by computing bivariate correlations using Pearson’s R tests. Hierarchical regression analysis was performed in order to assess the impact of the respective TPB measures as predictors on the intention to perform direct observations.

**Results:**

In our study 82 GP supervisors completed our TPB questionnaire. We found that supervisors had a positive attitude towards direct observations. Our TPB model explained 45% of the variance in supervisors’ intentions to perform them. Normative beliefs and past behaviour were significant determinants of this intention.

**Conclusion:**

Our study suggests that supervisors use their past experiences to form intentions to perform direct observations in a careful, thoughtful manner and, in doing so, also take the preferences of the learner and other stakeholders potentially engaged in direct observations into consideration. These findings have potential implications for research into work-based assessments and the development of training interventions to foster a shared mental model on the use of direct observations.

## Background

Direct observation (DO) by supervisors of trainee clinical task performance plays a pivotal role in competency-based medical education [1]. DO is a prerequisite for both high-quality learning and robust decision-making on a learner’s competence development [2]. It can serve as a method to monitor trainee day-to-day performance and provide supervisors with important information that can be used to give feedback on a variety of competencies required for safe patient care [3–8]. Hence, DO not only serves as an indispensable tool to warrant the quality of patient care, it also enables decisions about levels of entrustment in task performance, to maximally foster trainee learning [9]. Consequently, incorporating DO in medical training programmes is strongly recommended [9, 10].

However, a substantial body of research has demonstrated that trainees are infrequently observed during clinical interactions with patients [4, 11–15]. Research findings point to a broad range of factors that potentially inhibit supervisors’ engagement in DO of clinical performance. First, as the workload in healthcare settings is typically high, supervisors may consider DOs of trainees time-consuming and inefficient [16]. Second, studies have shown that supervisors’ initiation of DO may conflict with trainees’ pursuit of independence and autonomy (being core values in medicine), emphasizing the role of socio-cultural factors in clinical education [17, 18]. Third, supervisors may feel that trainees alter their behaviour during the observation, thereby raising concerns that DOs assess the ‘shows how’ level rather than what trainees actually ‘do’ in clinical practice [19, 20]. Finally, recent research has suggested that supervisors may perceive low self-efficacy in performing observations of clinical performance and the provision of feedback [19, 21].

Altogether, although formal guidelines encourage and maybe even require supervisors to perform DOs, practical, socio-cultural as well as personal barriers may underlie the lack of DO reported in the medical education literature. To better understand supervisors’ actual performance of DOs, we set out to study supervisors’ behavioural intention to perform DO. Intention as a key determinant of action has proved invaluable for researchers concerned with behaviour and behavioural change. Numerous correlational studies in different fields have indicated that intentions predict actual behaviour [22–25]. The Theory of Planned Behaviour (TPB) [24] has been used extensively and successfully to investigate the relations between behavioural intentions and its underlying beliefs in the fields of health promotion [26–29], patient care [30–34] and medical education [35–37]. In this study, the TPP will be applied to systematically examine and understand the factors associated with supervisors’ intention to observe trainees in the clinical workplace. Findings may thereby support the implementation of guidelines in order to increase the use of DO.

## Methods

The TPB proposes that human behaviour is guided by three categories of beliefs: behavioural, normative and control beliefs. Behavioural beliefs are about the perceived consequences of the behaviour. These beliefs influence one’s attitude towards the respective behaviour either positively or negatively. Normative beliefs are about expectations from other people, resulting in perceived, subjective social norms. Control beliefs are about the presence of factors that may facilitate or inhibit intended behaviours, giving rise to perceived behavioural control. The result of these three categories of beliefs combined, that is, attitude towards the respective behaviour, subjective norms and perceived behavioural control, leads to the formation of a behavioural intention.

Our application of the TPB followed the five consecutive stages described in the manual by Francis and colleagues (2004): [1] definition of the behaviour of interest [2]; identification of participants and context [3]; instrument development: TPB questionnaire item generation [4]; data collection; and [5] statistical analysis [38].

### Definition of the behaviour of interest

Using the TACT (Target, Action, Context and Time) principle, we defined the behaviour of interest as: performing direct real-time observations of trainees and providing feedback by supervisors during workplace-based medical residency training [39].

### Identification of participants and context

Participants were General Practice (GP) supervisors from two GP specialty training institutes in the Netherlands (Maastricht and Leiden). During years 1 and 3 of the three-year postgraduate GP training programme, trainees spend4 days per week in general practice where a GP supervisor monitors, coaches and assesses their competence development.

### Instrument development: TPB questionnaire item generation

**Fig. 1:**
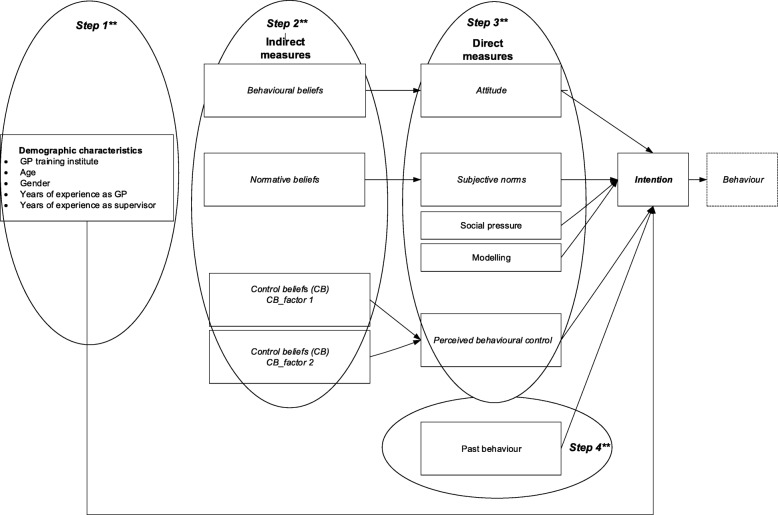
Extended* TPB model for the prediction of intention of supervisors to perform direct observations in the clinical workplace. *The original TPB model (*in italics*) is extended by the ‘Social pressure’,’ Modelling’ and ‘Past behaviour’ measures. **We performed these steps in the hierarchical regression analysis described in the Results section

Our 56-item web-based questionnaire (Additional file 1) consisted of three parts, namely a general introduction that contained the definition of the behaviour of interest, five items on demographic variables and 51 TPB statements. In line with recommendations, questionnaire items addressed respondents’ attitude, subjective norms and perceived behavioural control (Fig. 1) both directly, by asking questions about their attitude, perceived norms and control in general, and indirectly, by asking questions about underlying specific beliefs [38, 39]. Beliefs emanated from three audio recorded focus groups with in total 21 GP supervisors [38, 40]. Data generated from the focus groups were transcribed verbatim and qualitatively coded with the aid of Nvivo software [41]. According to principles of qualitative data analysis, three researchers (AT, MG, LJ) independently categorized codes into themes and used the belief categories of the TPB (i.e. behavioural, normative, control) as a preliminary coding framework. Discrepancies in the coding process were resolved through constant comparison and discussion within the research team [42].After that, themes were listed in order of frequency. These themes were used to generate questionnaire items in order for the final TPB questionnaire to cover 75% of the cumulative frequency of all beliefs that were reported in the focus groups [39]. As recommended, the behavioural and normative beliefs were converted into two types of items; one set of statements about behavioural beliefs and corresponding behavioural outcome evaluations and one set about normative beliefs and corresponding motivation to comply [38]. Congruent with previous research studies, for instance by de Vries et al. [43], we utilised Bandura’s measures of self-efficacy to operationalise control beliefs within the TPB questionnaire [43, 44].

Since extensive research has demonstrated that past behaviour has a residual effect on intentions after controlling for other TPB measures, we chose to include ‘past behaviour’ as a determinant of the intention to perform DOs [24, 45–47]. Likewise, we included perceived social pressure (a perceived urge to adopt a behaviour) and modelling (seeing others perform a behaviour) as these factors may influence people’s intention to engage in the respective behaviour, extending the impact of subjective norms [48]. With the addition of these extra measures we composed an extended TPB model to explore and predict supervisors’ intentions to engage in DOs of trainee performance in the clinical workplace (Fig. 1).

All items were assessed on 7-point Likert scales and had defined anchors at the extremes (e.g. good-bad) [38]. The only exception were the control beliefs, and as recommended by Bandura (2006), these were rated on a 100-point scale ranging from 1 (great uncertainty) to 100 (complete certainty) with 10-unit intervals [49].

All members of the research team pre-tested a preliminary 65-item questionnaire for clarity, understanding, applicability and feasibility. Items were rephrased and when necessary deleted until no new recommendations for improvement were given. Next, the 56-item questionnaire was pilot tested by 10 GP supervisors, following which two more items were rephrased.

### Data collection

We used consecutive sampling from a list of 472 supervisors (all active GP supervisors of the institutes in Maastricht and Leiden) to obtain 200 potential respondents [38]. They were invited by email to complete the web-based TPB questionnaire between June and October 2017. Non-responders received email reminders after two and 4 weeks. The data collection period ended 2 months after the questionnaire was first emailed.

### Statistical analysis

Since one questionnaire form had two missing values, we replaced these with the respondent’s mean score for the remaining items within that measure. Where applicable, negatively keyed items were reverse coded to ensure that all items were in the same direction [38].

We calculated descriptive statistics for the following demographic characteristics: age, gender and years of work experience as a practising GP and GP supervisor (see Table 1). For each of the indirect and direct measures of ‘attitude’, ‘subjective norms’, ‘perceived behavioural control’ and ‘intention’we calculated item-to-total correlations with the goal of eliminating items that were not related to the same measure. Following item elimination, we estimated the internal consistency (Cronbach’s coefficient α) of the direct measures; an α of > 0.60 was considered as acceptable [38]. We subsequently calculated the means and standard deviations of the composite scores regarding the direct measures of attitude, subjective norms, perceived behavioural control and intention [38].

**Table 1:** Demographic variables of GP supervisors (*N* = 82)

Demographic variable	N (%)
*Training institute*	
Maastricht	49 (60)
Leiden	33 (40)
*Age (years)*	
31–40	8 (10)
41–50	33 (40)
51–65	41 (50)
*Gender*	
Male	47 (57)
Female	35 (43)
*Experience as GP*	
6–10 years	7 (9)
> 10 years	75 (91)
*Experience as supervisor*	
0–5 years	23 (28)
6–10 years	33 (40)
> 10 years	26 (32)

We did not perform a reliability analysis of the indirect, belief-based measures as Ajzen [24] stated that internal consistency is not a necessary feature of these measures because ‘beliefs towards a behaviour can be ambivalent when a behaviour is likely to produce both positive and negative outcomes’. As the ‘control beliefs’ measure consisted of 12 items, and self-efficacy is, according to Bandura, a multifaceted concept [49], we performed an exploratory factor analysis to check for potential separate intercorrelated subscales. We used an oblimin rotation (delta = 0) in order to optimise the interrelated pattern of factor loadings of the control belief items [50]. According to the guidelines, the criteria for factor loading cut-offs were > 0.5 (good), > 0.6 (very good) and > 0.7 (excellent) [51]. Identified subscales were treated as distinct measures in the analysis. For the analysis of the other indirect, belief-based measures we defined composite scores: we weighted (multiplied) each behavioural belief by the corresponding score for outcome evaluation and each normative belief by the corresponding score for motivation to comply. Finally, we summed the weighted beliefs to create a composite score for the behavioural and normative beliefs respectively [38].. As the TPB model contains theoretical measures that are assumed to be interrelated, we also explored the relationships between the TPB measures of our questionnaire by computing bivariate correlations using Pearson’s R tests.

In order to assess the impact of the respective TPB measures as predictors on the intention to perform DOs, we did a hierarchical regression analysis [39]. We checked the assumptions for linear regression analysis (linearity, independence, normal distribution and equal variance of residuals [51]). We subsequently calculated standardised beta weights to examine the contribution of the different predictors to the regression equation. As a first step, the demographic variables were entered into the model, followed by the indirect measurements of ‘attitude’, ‘norms’ and ‘control beliefs’ at step two (see Fig. 1). In the third step, we added the direct measurements of ‘attitude’, ‘subjective norms’, ‘social pressure’, ‘modelling’ and ‘perceived behavioural control’. Finally, we entered the ‘past behaviour’ measure into the regression equation. We performed all statistical analyses using SPSS, version 25 [52].

## Results

The net response rate was 41% (*N* = 82). As can be inferred from Table 1, presenting the descriptive demographic features of participants, our sample had a relatively equal distribution of age, gender and training institute. Additionally, 78% of participants reported that they had performed more than three DOs over the past three-month period.

### Assessing the structure of the measures in the TPB-questionnaire

Based on reliability analyses of the direct measures of the TPB questionnaire, the Cronbach’s α values were 0.92 for intention, 0.75 for subjective norms, 0.73 for attitude, and 0.65 for perceived behavioural control, indicating that the internal consistency values ranged from satisfactory to very good across our sample [38]. Scores for the control belief [1] items were normally distributed among the questionnaire scores, a precondition for using the maximum likelihood extraction method in the exploratory factor analysis [50]. A scree plot of the extracted factors pointed to the presence of two separate factors in the data, with Eigenvalues of 4.0 and 1.8 respectively. CB factor 1 contained four suitable loadings and CB factor 2 contained five suitable loadings (Table 2). These two factors were iteratively interpreted by four team members (AT, MG, IM, LJ). The best fitting descriptive label for CB factor 1 (4 items) was found to be ‘self-confidence in performing the task of DO’ and for CB factor 2 (5 items) ‘feelings of stress induced by practical conditions that limit the performance of DOs’. We treated these two factors as distinct measures in the analysis of the TPB questionnaire.

**Table 2:** Exploratory factor analysis of control beliefs in the TPB model

Control beliefs	Factor loadings*	
	CB factor 1	CB factor 2
*I can perform direct observations of the trainee when/if…*		
I am busy	−.047	**.721**
I am not feeling comfortable	−.031	**.636**
Trainees have a specific question for feedback	.108	**.536**
Trainees avoid direct observations	.265	**.532**
Clear assessment criteria are available to perform direct observations	−.077	**.502**
I feel the need to give a critical assessment	.266	.211
I am trained in performing direct observations	.405	.259
I have the feeling that performing direct observations is disturbing the contact between trainee and patients	.435	.087
I do not know what to assess	**.703**	.048
I have the feeling that my roles as supervisor and assessor are in conflict	**.704**	−.100
I have the feeling that a trainee experiences direct observations as an assessment	**.746**	−.040
I have the feeling that I am confronted with shortcomings in my own consultations	.791	−.111

*Significant factor loadings in **bold**

### Determinants of supervisors’ intention to perform DOs

The means, standard deviations and correlations between the extended TPB measures are presented in Table 3. On average, participants reported a strong intention to perform DOs, and a positive attitude, positively perceived subjective norms and a relatively high perceived behavioural control towards performing DOs (all mean scores above five on a seven-point scale, Table 3). A significant and positive correlation between intention and all indirect and direct measures in the TPB model was revealed. Furthermore, the indirect, belief-based measures were all positively and significantly correlated with their corresponding direct measurements. Likewise there was a significant correlation between control beliefs (total) and its direct measure ‘perceived behavioural control’(r = .33, *p* < 0.01). But, unlike CB factor 2 (‘feelings of stress induced by practical conditions that limit the performance of DOs’, r = .56, *p* < 0.01), CB factor 1 (‘self-confidence in performing the task of DO’) had no significant correlation with perceived behavioural control (r = .06, ns). From the additional measures only past behaviour had a significant correlation with intention; there was no significant correlation between intention and the measures ‘social pressure’ and ‘modelling’. However, there was a positive correlation between modelling and normative beliefs and a negative correlation between social pressure and supervisors attitude towards performing DOs.

**Table 3:** Correlation matrix and descriptive statistics for measures of the extended TPB model

*N = 82*	1.	2.	3.	4.	5.	6.	7.	8.	9.	10.	11.	Mean	SD	Range
1. Intention												6.62	.81	1–7
2. Behavioural beliefs	.43**											5.03	.70	1–7
3. Normative beliefs	.31**	.39**										4.73	1.0	1–7
4. Control beliefs (total)	.38**	.44**	.12									54.66	16.02	0–100
5. CB factor 1^1^	.23*	.28**	.13	.81**								50.54	24.15	0–100
6. CB factor 2^2^	.42**	.49**	.06	.76**	.32**							54.66	18.35	0–100
7. Attitude	.32**	.47**	.23*	.22*	.05	.38**						5.33	1.31	1–7
8. Subjective norms	.36**	.33**	.38**	.14	.20	.23*	.23*					5.35	1.15	1–7
9. Social pressure	.0	.24*	.15	−.10	−.03	−.22*	−35**	.21				3.40	1.77	1–7
10. Modelling	.08	−.05	.42**	−.08	−.05	−.12	.02	.17	.09			4.83	1.16	1–7
11. PBC^3^	.36**	.33**	−.04	.33**	.06	.56**	.33**	.10	−37**	−.02		6.15	.70	1–7
12. Past behaviour	.37**	.09	−.05	−.01	−.06	.14	.07	.43**	.05	.02	.15	78^4^		

Numbers in the first horizontal row correspond with the numbers and labels in the first vertical column

^1^CB factor 1: control beliefs factor 1; ^2^CB factor 2: control beliefs factor 2; ^3^PBC: perceived behavioural control; ^4^% of supervisors performing more than three direct observations over the past three-month period; ** Correlation significant at 0.01 level;* Correlation significant at 0.05 level

### Predicting supervisors’ intention to perform DOs

We conducted a four-step hierarchical regression analysis to test the impact of the measures of the extended TPB model on the prediction of supervisors’ intention to perform DOs (Table 4). In the first step of the analysis, we entered the demographic variables into the predictive equation. The demographic variables did not account for a statistically significant proportion of the variance in intention (R^2^_change_ = 0.02, F(5,76) = 0.37, *p* = 0.87). Entry of the indirect measures of the TPB in step 2 resulted in a statistically significant increase in the variance explained (R^2^_change_ = 0.29, F_change_ (4,72) =3.56, *p* < 0.01). Normative beliefs (i.e. beliefs about the normative expectations of other people) and CB factor 2 (i.e. feelings of stress induced by practical conditions that limit the performance of DOs) had a significant positive beta weight of .30 and .25 respectively, reflecting their contribution to the predictive equation. Behavioural beliefs (β = .18) and CB factor 1 (β = .03) were not significantly related to intention. At step 3, the addition of the direct measures resulted in a further increase in the variance explained (R^2^_change_ = 0.8, F_change_ (5,67) = 2.76, *p* = .31); the weighted betas of all single measures were no longer significant, indicating no single measure had a significant contribution in the predictive equation of the intention to perform DOs. At step 4, entry of the additional variable ‘past behaviour’ resulted in a further statistically significant increase of 8% in the variance explained to 45% (R^2^_change_ = 0.8, F_change_ (1,66) =3.57, *p* = .003). Moreover, both normative beliefs (β = .27, *P* < 0.05) and past behaviour (β = .33, *p* < 0.01) had a statistically significant beta weight.

**Table 4:** Determinants of the intention to perform direct observations resulting from a hierarchical regression analysis (*N* = 82)

Step	Determinants	R^2^	R^2^_change_	F_change_	Standardised betas			
					Step 1	Step 2	Step 3	Step 4
1.	*Demographics*	*.02*	.024	.37				
	Training institute				.01	.44	.08	.10
	Age				.02	−.07	−.03	−.01
	Gender				−.11	−.12	−.03	−.04
	Experience as GP				.08	.17	.11	.11
	Experience as supervisor				−.14	−.06	−.08	−.07
2.	*Indirect measures*	.31	.29	7.43**				
	Behavioural beliefs					.18	.12	.11
	Normative beliefs					.25*	.19	.27*
	CB Factor 1					.03	.09	.10
	CB Factor 2					.30*	.13	.13
	*Direct measures*	.37	.08	1.21				
	Attitude						.09	.11
	Subjective norm						.14	−.01
	Social pressure						.12	.12
	Modelling						−.01	−.01
	PBC^1^						.26	.22
	*Additional variables*							
	Past behaviour	.45	.08	9.76**				.33**

^1^PBC: perceived behavioural control; ** correlation significant at 0.01 level;*correlation significant at 0.05 level

## Discussion

Our findings suggest that the extension of the TPB model with the past behaviour measure enabled our understanding of the determinants that may influence supervisors’ intention to perform DOs. Our model appeared to explain 45% of the variance in supervisors’ behavioural intentions, which is consistent with findings from meta-analytic reviews on the efficacy of the TPB [25, 53]. Besides past behaviour, normative beliefs emerged as a significant determinant of supervisors’ intention to perform DOs. Furthermore, consistent with the theoretical framework of the TPB, we found a significant and positive correlation between intention and all indirect and direct measures in the TPB model. The indirect, belief-based measures were all positively and significantly correlated with their corresponding direct measures, thereby confirming the validity of the indirect measures in our TPB model.

An important finding from our study was the significant contribution of ‘past behaviour’ to supervisors’ intention to perform DOs. Several TPB studies have found that past behaviour helps to predict the intention to exhibit future behaviour, even after all determinants of the TPB model have been accounted for [24, 25, 45, 46, 54]. Ouelette et al. (1998) suggested that there are two potential routes through which past behaviour affects future behaviour [46]. The first is through performance in stable and predictable settings, where behaviour can become automatic and habitual. In such situations, the frequency of past behaviour reflects habit strength and has a direct effect on future behaviour [46]. Examples of such behaviours are coffee consumption or seat belt use. The second route is more applicable to the behaviour of interest in our study, that is, performing DOs in the typically unpredictable and complex setting of clinical practice. This route requires deliberative reasoning to initiate and display the behaviour. Ouelette et al. (1998) found that in domains that encouraged deliberatively guided behaviour, beliefs about other people’s normative expectations had a strong impact on intentions [46]. These results echo our findings that both past behaviour and normative beliefs are significant determinants of supervisors’ intention to perform DOs.

In our study, supervisors’ beliefs about the normative expectations of other people appeared to be a more important determinant of the intention to engage in DOs than beliefs about the consequences and beliefs about self-confidence or controllability. Our measurement of normative beliefs specifically reflected supervisors strongly feel that learners, supervisor colleagues, residency training institute and patients expect them to engage in DOs. This finding is supported by a positive correlation between modelling (perceiving that other supervisors perform DOs) and normative beliefs. Furthermore, it is in line with the negative correlation of perceived social pressure with supervisors attitude towards performing DOs, which may express that supervisors consider themselves as highly autonomous functioning professionals, both in GP practice and in the one-on-one training of trainees. Our findings resonate with TPB studies in other fields showing that behaviours that have potential implications for others -as well as self- are influenced more by normative beliefs than by behavioural beliefs. Kortteisto et al.(2010), for example, found that normative beliefs were the most important factor inducing nurses to use clinical guidelines when making treatment decisions [30]. Similar results were reported in a study by Steadman et al. (2002) on attendance at preventive screening [55].

### Strengths and limitations

To our knowledge, this is the first theory-driven study to explore and predict supervisors’ intention to engage in DOs in a complex clinical setting using a theory-informed (TPB) questionnaire [38, 39].

Several limitations need addressing. First, the response rate of 41% was moderate, a common challenge in questionnaire studies [38, 56]. The distribution of participants’ demographic features was similar to those found in other GP specialty training institutes in the Netherlands [57]. We had to include at least 40 items in our questionnaire as this is considered a minimum for TPB research [40]. The incorporation of additional measures (past behaviour, social influence and additional control belief items) led to a questionnaire that comprised 56 items. Our survey length may have influenced respondents’ acceptance of the questionnaire since response rate tends to be negatively correlated with the number of items used [56]. Despite the modest response rate, however, a sample size of over 80 participants is deemed acceptable in TPB research [38, 51]. Second, we conducted our study in a postgraduate medical specialty training setting characterised by long-term one-on-one relationships between supervisors and trainees. However, the transferability of the present findings to other medical specialties and work-based learning settings may be restricted. Hospital-based supervisors, in contrast, typically have short-term contacts with multiple trainees who potentially have different normative expectations that must be met. Although in these circumstances it is equally important that a shared understanding of the role of DOs be fostered, normative beliefs may have a less significant effect on supervisors’ intention to perform DOs. Finally, recently authors addressed some limitations on the use of the TPB [58, 59]. Although behavioural intention has been shown to be a valid proxy measure of actual behaviour [22–25], we measured intentions rather than actual behaviour. In this respect, the TPB is a continuum model in which influential predictor variables are typically combined into one linear prediction equation that places individuals along a continuum of behaviour likelihood, in our case supervisors performance of DOs. However, continuum models typically do not account for the postintentional phase in which goals are translated into action [59]. Future research addressing postintentional beliefs may provide further insight in the relationship between intention and actual behaviour. In addition, information on the frequency of supervisors performing DOs in our setting is lacking. Consequently, we were not able to use our extended TPB model to explore the intention-behaviour correlation, nor to predict actual behaviour yet, which remains a challenge for future research.

### Implications for practice and research

Our findings show that, in the complex context of postgraduate training for general practice, supervisors use their past experiences to develop intentions in a thoughtful manner and, in doing so, also take the preferences of the learner and other stakeholders in residency training into account. Supervisors are more inclined to engage in DO if they feel that others, among which learners, expect DO to be part of residency training. As a consequence, supervisors’ intention to perform DOs may rely on learners’ initiative to explicitly ask for it. However, several studies have suggested that learners are ambivalent about being observed. Reasons for this ambivalence as reported in the literature are that DOs may conflict with their pursuit of independence and autonomy and that the (formative and/or summative) purpose of the observation is not always clear [17, 18, 60, 61]. Therefore, both supervisor and learner need a clear, articulated and shared perspective on the role and use of DOs. By discussing and clarifying underlying assumptions and beliefs, they may be able to overcome potential barriers to the use of DOs and develop a shared understanding of the role of DOs in learning and assessment. Our findings illustrate that the training institute may also influence supervisors’ intention to perform DOs. To improve observations of clinical performance but also to foster a shared mental model on the use of DOs in postgraduate medical training programmes, we recommend that training institutes articulate their expectations regarding supervisors’ engagement in DOs more clearly, explicitly and consistently. By translating their normative expectations into ongoing training and coaching of supervisors, training institutes may effectively contribute to the enactment of supervisor intentions into actual performance of DOs. Such regular training should not only include sessions on how to effectively conduct DOs but also provide guidance as to how to foster the shared responsibility of learner and supervisor in planning them [62]. Similarly, our results emphasise the role of supervisor colleagues in promoting DOs in residency training. DO group training with supervisor colleagues may therefore not only improve supervisors’ technical skills and self-efficacy regarding the performance of DOs, it may also encourage supervisors to perform DOs more frequently by making them part of a community of supervisors with similar roles and tasks [63, 64].

Our exploratory quantitative approach has yielded useful directions for further research on DOs and other deliberately guided behaviours. Such research, and pedagogical action research in particular, may more clearly delineate the impact of normative expectations of various stakeholders on supervisors’ use of DOs. Accurate registration of DO frequency in daily practice is needed to explore the link between supervisors’ intention to perform DOs and their actual behaviours and to study the efficiency of training interventions.

## Conclusion

This study helps to expand current knowledge in the emerging field of workplace based learning in clinical practice. It enhances our understanding of the processes inhibiting and facilitating supervisors’ intention to perform DOs. Understanding different expectations regarding the use of DOs could be an important step to increase the frequency of DOs and in developing a shared mental model on their use in workplace-based learning.

## Supplementary information

**Additional file 1.** Translated questionnaire (Direct observations during workplace-based medical residency training)

## Data Availability

The dataset generated and analysed during the current study is available from the corresponding author on request.

## Abbreviations

CBME: Competency-based medical education
DO: Direct Observations
GP: General Practice
TPB: Theory of Planned Behaviour

## References

[1] Swing SR, International CC (2010). Perspectives on competency-based medical education from the learning sciences. Med Teach.

[2] Hauer KE, Holmboe ES, Kogan JR (2011). Twelve tips for implementing tools for direct observation of medical trainees' clinical skills during patient encounters. Med Teach..

[3] Pelgrim EA, Kramer AW, Mokkink HG, van den Elsen L, Grol RP, van der Vleuten CP (2011). In-training assessment using direct observation of single-patient encounters: a literature review. Adv Health Sci Educ Theory Pract.

[4] Holmboe ES (2004). Faculty and the observation of trainees' clinical skills: problems and opportunities. Acad Med.

[5] De Haes JC, Oort FJ, Hulsman RL (2005). Summative assessment of medical students' communication skills and professional attitudes through observation in clinical practice. Med Teach..

[6] Dorfsman ML, Wolfson AB (2009). Direct observation of residents in the emergency department: a structured educational program. Acad Emerg Med.

[7] Stojan JN, Clay MA, Lypson ML. Assessing patient-centred care through direct observation of clinical encounters. BMJ Qual Saf. 2015. 10.1136/bmjqs-2015-004584 26424761

[8] Wendling AL (2004). Assessing resident competency in an outpatient setting. Fam Med.

[9] Holmboe ES (2015). Realizing the promise of competency-based medical education. Acad Med.

[10] Carraccio CL, Englander R (2013). From Flexner to competencies: reflections on a decade and the journey ahead. Acad Med.

[11] Burdick WP, Schoffstall J (1995). Observation of emergency medicine residents at the bedside: how often does it happen?. Acad Emerg Med.

[12] Chen W, Liao SC, Tsai CH, Huang CC, Lin CC, Tsai CH (2008). Clinical skills in final-year medical students: the relationship between self-reported confidence and direct observation by faculty or residents. Ann Acad Med Singap.

[13] Howley LD, Wilson WG (2004). Direct observation of students during clerkship rotations: a multiyear descriptive study. Acad Med.

[14] Day SC, Grosso LJ, Norcini JJ, Blank LL, Swanson DB, Horne MH (1990). Residents' perception of evaluation procedures used by their training program. J Gen Intern Med.

[15] Norcini J, Burch V (2007). Workplace-based assessment as an educational tool: AMEE guide no. 31. Med Teach..

[16] Madan R, Conn D, Dubo E, Voore P, Wiesenfeld L (2012). The enablers and barriers to the use of direct observation of trainee clinical skills by supervising faculty in a psychiatry residency program. Can J Psychiatr.

[17] Kennedy TJ, Regehr G, Baker GR, Lingard LA (2009). 'It's a cultural expectation...' The p ressure on medical trainees to work independently in clinical practice. Med Educ.

[18] Watling C, LaDonna KA, Lingard L, Voyer S, Hatala R (2016). 'Sometimes the work just needs to be done': socio-cultural influences on direct observation in medical training. Med Educ.

[19] Kirby J, Archibeque L, Confer L, Baird D (2016). Workplace formative assessment: faculty members' beliefs. Clin Teach.

[20] Miller GE (1990). The assessment of clinical skills/competence/performance. Acad Med.

[21] Kogan JR, Conforti LN, Bernabeo EC, Durning SJ, Hauer KE, Holmboe ES (2012). Faculty staff perceptions of feedback to residents after direct observation of clinical skills. Med Educ.

[22] Sheeran P (2002). Intention—behavior relations: a conceptual and empirical review. Eur Rev Soc Psychol.

[23] Webb TL, Sheeran P (2006). Does changing behavioral intentions engender behavior change? A meta-analysis of the experimental evidence. Psychol Bull.

[24] Ajzen I (1991). The theory of planned behavior. Organ Behav Hum Dec.

[25] McEachan RRC, Conner M, Taylor NJ, Lawton RJ (2011). Prospective prediction of health-related behaviours with the theory of planned behaviour: a meta-analysis. Health Psychol Rev.

[26] Dunstan DA, Covic T, Tyson GA (2013). What leads to the expectation to return to work? I nsights from a theory of planned behavior (TPB) model of future work outcomes. Work..

[27] Chorlton K, Conner M, Jamson S (2012). Identifying the psychological determinants of risky riding: an application of an extended theory of planned behaviour. Accid Anal Prev.

[28] Jones SC (2016). Parental provision of alcohol: a TPB-framed review of the literature. Health Promot Int.

[29] Godin G, Kok G (1996). The theory of planned behavior: a review of its applications to health-related behaviors. Am J Health Promot.

[30] Kortteisto T, Kaila M, Komulainen J, Mantyranta T, Rissanen P (2010). Healthcare professionals' intentions to use clinical guidelines: a survey using the theory of planned behaviour. Implement Sci.

[31] Mtenga SM, Exavery A, Kakoko D, Geubbels E (2015). Social cognitive determinants of HIV voluntary counselling and testing uptake among married individuals in Dar Es Salaam Tanzania: theory of planned behaviour (TPB). BMC Public Health.

[32] Perez R, Brehaut JC, Taljaard M, Stiell IG, Clement CM, Grimshaw J (2014). Theory of planned behaviour can help understand processes underlying the use of two emergency medicine diagnostic imaging rules. Implement Sci.

[33] Tsiantou V, Shea S, Martinez L, Agius D, Basak O, Faresjo T (2013). Eliciting general practitioners' salient beliefs towards prescribing: a qualitative study based on the theory of planned behaviour in Greece. J Clin Pharm Ther.

[34] Hung SY, Ku YC, Chien JC (2012). Understanding physicians' acceptance of the Medline system for practicing evidence-based medicine: a decomposed TPB model. Int J Med Inform.

[35] Tian J, Atkinson NL, Portnoy B, Lowitt NR (2010). The development of a theory-based instrument to evaluate the effectiveness of continuing medical education. Acad Med.

[36] Archer R, Elder W, Hustedde C, Milam A, Joyce J (2008). The theory of planned behaviour in medical education: a model for integrating professionalism training. Med Educ.

[37] Hadadgar A, Changiz T, Masiello I, Dehghani Z, Mirshahzadeh N, Zary N (2016). Applicability of the theory of planned behavior in explaining the general practitioners eLearning use in continuing medical education. BMC Med Educ..

[38] Francis J, Eccles, MP., Johnston, M., Walker, AE., Grimshaw, JM., Foy, R., Kaner EFS, Smith, L. & Bonetti, D. Constructing questionnaires based on the theory of planned behaviour: A manual for health services researchers. Newcastle upon Tyne, UK: Centre for Health Services Research, University of Newcastle upon Tyne.2004.

[39] Ajzen I. Constructing a TpB questionnaire: Conceptual and Methodological Considerations 2006 [Available from: https://people.umass.edu/aizen/pdf/tpb.measurement.pdf.

[40] Oluka OC, Nie S, Sun Y (2014). Quality assessment of TPB-based questionnaires: a systematic review. PLoS One.

[41] Nvivo. NVivo qualitative data analysis Software; QSR International Pty Ltd. Version 11, 2016.

[42] Lingard L, Albert M, Levinson W (2008). Grounded theory, mixed methods, and action research. BMJ..

[43] de Vries H, Dijkstra M, Kuhlman P (1988). Self-efficacy: the third factor besides attitude and subjective norm as a predictor of behavioural intentions. Health Educ Res.

[44] Bandura A (1977). Self-efficacy: toward a unifying theory of behavioral change. Psychol Rev.

[45] Conner M, Armitage CJ (1998). Extending the theory of planned behavior: a review and avenues for further research. J Appl Soc Psychol.

[46] Ouellette JA, Wood W (1998). Habit and intention in everyday life: the multiple processes by which past behavior predicts future behavior. Psychol Bull.

[47] Fishbein M, Ajzen I. Predicting and changing behavior : Challenges to the reasoned action approach. New York: Psychology Press; 2010. xix, 518 p. p.

[48] de Vries H, Backbier E, Kok G, Dijkstra M. The Impact of Social Influences in the Context of Attitude, Self-Efficacy, Intention, and Previous Behavior as Predictors of Smoking Onset 11995. 237–57 p.

[49] Bandura A, Urdan T, Pajares F (2006). Guide for constructing self-efficacy scales. Self-efficacy beliefs of adolescents.

[50] Costello A. Best Practices in Exploratory Factor Analysis: Four Recommendations for Getting the Most From Your Analysis. Practical Assessment, Research & Evaluation. 2005;10(7):Available online: http://pareonline.net/getvn.asp?v=10&n=7.

[51] Tabachnick BG, Fidell LS. Using multivariate statistics. 6th ed. Boston: Pearson Education; 2013. xxxi, 983 p. p.

[52] IBM Corp. Released 2017. IBM SPSS statistics for windows, version 25.0. Armonk, NY: IBM Corp.

[53] Armitage CJ, Conner M (2001). Efficacy of the theory of planned behaviour: a meta-analytic review. Br J Soc Psychol.

[54] Ajzen I (2011). The theory of planned behaviour: reactions and reflections. Psychol Health.

[55] Steadman L, Rutter DR, Field S (2002). Individually elicited versus modal normative beliefs in predicting attendance at breast screening: Examining the role of belief salience in the Theory of Planned Behaviour. Br J Health Psychol.

[56] Edwards PJ, Roberts I, Clarke MJ, Diguiseppi C, Wentz R, Kwan I (2009). Methods to increase response to postal and electronic questionnaires. Cochrane Database Syst Rev.

[57] Esser K. SBOH Sociaal jaarverslag 2017 2017 [Available from: https://www.sboh.nl/images/bestanden/Algemeen/Organisatie/jaarverslag_2017_compleet_definitief_180614.pdf.

[58] Sniehotta FF, Presseau J, Araújo-Soares V (2014). Time to retire the theory of planned behaviour. Health Psychol Rev.

[59] Schwarzer R (2008). Modeling health behavior change: how to predict and modify the adoption and maintenance of health behaviors. Appl Psychol.

[60] Teunissen PW, Stapel DA, van der Vleuten C, Scherpbier A, Boor K, Scheele F (2009). Who wants feedback? An investigation of the variables influencing residents' feedback-seeking behavior in relation to night shifts. Acad Med.

[61] Bok HG, Teunissen PW, Favier RP, Rietbroek NJ, Theyse LF, Brommer H (2013). Programmatic assessment of competency-based workplace learning: when theory meets practice. BMC Med Educ.

[62] Rietmeijer CBT, Huisman D, Blankenstein AH, de Vries H, Scheele F, Kramer AWM, et al. Patterns of direct observation and their impact during residency: general practice supervisors' views. Med Educ. 2018. 10.1111/medu.13631 PMC6120450 30043397

[63] Kogan JR, Conforti LN, Bernabeo E, Iobst W, Holmboe E (2015). How faculty members experience workplace-based assessment rater training: a qualitative study. Med Educ.

[64] Holmboe ES, Hawkins RE, Huot SJ (2004). Effects of training in direct observation of medical residents' clinical competence: a randomized trial. Ann Intern Med.

