# Supplementary material for: Supervisors’ intention to observe clinical task performance: an exploratory study using the theory of planned behaviour during postgraduate medical training
Source: BMC Med Educ. 2020 Apr 30;20:134. doi: 10.1186/s12909-020-02047-y (PMC7193388; doi:10.1186/s12909-020-02047-y)
Supplement: Supplementary file 1 — Additional file 1. Translated questionnaire (Direct observations during workplace-based medical residency training) [file 12909_2020_2047_MOESM1_ESM.docx]

**Additional file 1**

**TPB Questionnaire: Direct observations during workplace-based medical residency training.**

**Demographic items**

1. In which region are you a clinical supervisor?

[Maastricht/Leiden]

1. What is your age?

[Number]

1. Are you male or female?

[Male/female]

1. How many years of experience do you have as a GP?

[5-10; more than 10 years]

1. How many years of experience do you have as a clinical supervisor?

[0-5; 5-10; more than 10 years]

**Past behaviour**

1. Can you estimate how often you performed a direct observation of your latest trainee in the past three months?

[0; 1-3; more than three times]

**General behavioural Intention**

Since you will be supervising a trainee in the clinical workplace in the three months’ to come,

1. are you willing to perform direct observations of your trainee?
2. do you intend to perform direct observations of your trainee?

most unlikely 1 2 3 4 5 6 7 Most likely

**Direct attitude towards direct observations**

*Performing direct observations of trainees in the clinical workplace is …*

9. not taxing 1 2 3 4 5 6 7 taxing

10. useful 1 2 3 4 5 6 7 useless

11. pleasant 1 2 3 4 5 6 7 unpleasant

**Direct perceived norms**

*As a clinical supervisor* …

1. most people who are important to me in my role as a supervisor think that I should perform direct observations
2. I am expected to perform direct observations
3. I experience social pressure to perform direct observations **(Social Pressure)**
4. I think that supervisor colleagues perform direct observations **(Modelling)**

strongly disagree 1 2 3 4 5 6 7 strongly agree

**Perceived behavioural control**

*As a clinical supervisor* …

1. I am confident that I can perform direct observations
2. I am capable of performing direct observations
3. I can decide whether or not I will perform direct observations

strongly disagree 1 2 3 4 5 6 7 strongly agree

**Outcome evaluations**

1. Insight into the competence development of my trainee is…
2. To disturb the contact between trainee and patient during consultations is …
3. To restrict the trainee’s autonomy is…
4. To contribute to more goal-oriented learning of the trainee is …
5. To provide specific feedback to the trainee is …
6. To improve the quality of my supervision of the trainee is …
7. Trainees asking for feedback more frequently is…
8. To gain educational experience as a supervisor is …

highly undesirable 1 2 3 4 5 6 7 highly desirable

**Behavioural beliefs**

*Performing direct observations of the trainee …*

1. provides me with insight into the competence development of the trainee
2. disturbs the contact between trainee and patient during consultations
3. restricts the trainee’s autonomy
4. contributes to more goal-oriented learning by the trainee
5. offers an opportunity to provide the trainee with specific feedback
6. improves the quality of my supervision of the trainee
7. results in trainees asking for feedback more frequently
8. is an educational experience for me as a supervisor

strongly disagree 1 2 3 4 5 6 7 strongly agree

**Normative beliefs**

1. A trainee expects me to perform direct observations
2. The vocational training institute expects me to perform direct observations
3. Health workers in my practice (nurses, practitioner colleagues) expect me to perform direct observations
4. Patients in my practice expect me to perform direct observations
5. Colleague supervisors expect me to perform direct observations

unlikely 1 2 3 4 5 6 7 likely

**Motivation to comply**

*It is important to know what…*

1. trainees expect from me
2. the vocational training institute expects from me
3. health workers in my practice (nurses, practitioner colleagues) expect from me
4. patients in my practice expect from me
5. supervisor colleagues expect from me

not at all 1 2 3 4 5 6 7 very much

**Control beliefs**

*I can perform direct observations of the trainee when/if …*

1. I am busy
2. clear assessment criteria are available to perform direct observations
3. trainees have a specific question for feedback
4. I am trained in performing direct observations
5. trainees avoid direct observations
6. I am not feeling comfortable
7. I feel the need to give a critical assessment
8. I have the feeling that my roles as supervisor and assessor are in conflict
9. I have the feeling that I am confronted with shortcomings in my own consultations
10. I have the feeling that performing direct observations is disturbing the contact between trainee and patients
11. I have the feeling that a trainee experiences direct observations as an assessment
12. I do not know what to assess

I certainly cannot 0 10 20 30 40 50 60 70 80 90 100 I certainly can
